# A Poultry Universal Primer-Based Fluorescent PCR (PUP-fPCR) for Simultaneous Identification and Quantification of Chicken, Quail, Duck, and Goose Meat Species

**DOI:** 10.3390/molecules31101590

**Published:** 2026-05-09

**Authors:** Yifan Li, Haoyang Cao, Guangxiang Chen, Xiaoyu Wang, Qiyue Yang, Mengyao Zhang, Jiaqi Yang, Rongyan Zhou, Wenjun Wang

**Affiliations:** 1 College of Animal Science and Technology, Hebei Agricultural University, Baoding 071000, China; 2 Experimental Training Center, Hebei Agricultural University, Baoding 071000, China

**Keywords:** comparative genomics, poultry universal primers, multiplex quantitative PCR, poultry species, meat adulteration

## Abstract

To combat poultry meat adulteration, we developed a poultry universal primer-based fluorescent PCR (PUP-fPCR). Through comprehensive genomic alignment analysis, a poultry-specific nuclear DNA sequence containing phylogenetically conserved regions and hypervariable segments with interspecies nucleotide polymorphisms was employed to develop universal primers targeting conserved flanking sequences and TaqMan probes for hypervariable segments. Then, a multiplex quantitative PCR method incorporating universal primers with four TaqMan probes was developed with high specificity and sensitivity (limit of detection: 0.005 ng). Analytical performance evaluation using prepared DNA mixtures revealed robust accuracy (relative deviation: 0.80–5.05%) and precision (relative standard deviation: 0.94–13.84%). This single-tube multiplex system leverages the spectral discrimination of TaqMan probes to simultaneously detect four poultry species, overcoming primer competition issues inherent in conventional multiplex PCR designs. This integrated approach reduces system complexity while maintaining detection efficiency, providing regulatory agencies with a robust tool for combating meat adulteration and ensuring food quality supervision.

## 1. Introduction

The growing complexity of the global food supply chain has thrust food safety and adulteration into the spotlight of critical global challenges. Many food authorities worldwide, such as the FDA, WHO, and the European Commission, have enacted distinct legislation addressing food fraud [1]. This has heightened consumer concerns regarding food safety and quality, thereby rendering food authenticity identification a pivotal research domain in food science. In 2020, China’s Ministry of Agriculture and Rural Affairs promulgated the “National Livestock and Poultry Genetic Resources Catalog”, officially designating 33 domesticated livestock and poultry species, among which chickens, ducks, and geese were included. Subsequently, in 2022, quail was incorporated into the National Catalog of Livestock and Poultry Genetic Resources as a type of traditional livestock and poultry, solidifying its position as the third-largest poultry industry in China, trailing only the chicken and duck industries. As common poultry species, chickens, quails, ducks, and geese exhibit significant similarities in morphological characteristics, genetic traits, and meat properties, making it difficult to effectively distinguish their meat products through visual inspection alone. However, significant disparities exist among these poultry meats in nutritional value, sensory flavor, and market price. Exploiting this combination of high similarity and substantial price gaps, unscrupulous merchants frequently engage in meat adulteration, substituting lower-priced poultry meat for higher-priced counterparts to illicitly maximize economic gains. Moreover, chicken and duck meat are also often used to pass off as pork or mutton [2,3]. This unethical practice not only disrupts market integrity and deceives consumers but also poses potential health risks [4], highlighting the urgent need for effective solutions to combat food adulteration.

Conventional approaches for meat adulteration detection primarily encompass lipid-, protein-, and DNA-based analytical techniques. Among these, DNA-based species identification methods have emerged as the most reliable detection strategy. This preference stems from the inherent limitations of protein and lipid biomarkers, which are susceptible to structural alterations during food processing and storage [5]. In contrast, DNA demonstrates superior thermal stability and chemical resilience [6], coupled with significant advantages in detection efficiency, cost-effectiveness, and sample processing [7]. Consequently, polymerase chain reaction (PCR) and its derivative technologies have been extensively employed for the identification of poultry-derived components in chicken and duck products. Such approaches include multiplex PCR [8,9], real-time PCR [10,11], and digital PCR [12,13], which enable qualitative or quantitative detection of species-specific components from chickens, ducks, and other species. Multiplex PCR, which employs multiple species-specific primer pairs to simultaneously amplify target DNA sequences, allows for the identification of multiple species within a single reaction system, thereby providing significant throughput advantages over singleplex PCR assays [14]. Notably, this approach is not without challenges. Excessive primers may induce cross-reactivity and increase the risk of primer-template mismatches [15], limiting the number of species that can be reliably identified in a single system. Additionally, the co-existence of template DNA and multiple primers within a single reaction further elevates the risk of non-specific amplification [16], potentially compromising assay accuracy. Real-time fluorescence PCR represents a technological advancement characterized by closed-tube detection, thereby eliminating post-amplification processing. Among these, TaqMan probe-based real-time PCR can prevent false-positive results and cross-contamination, while maintaining high specificity, high sensitivity, and operational simplicity, with performance superior to conventional PCR [17]. A multiplex TaqMan probe-based real-time PCR assay perfectly combines the advantages of multiplex PCR and real-time PCR, enabling simultaneous qualitative and quantitative analysis of multiple targets, including poultry. For example, a TaqMan-based triple real-time PCR assay with an endogenous reference gene has been developed for the detection of chicken, duck, and goose DNA in meat products, showing enhanced specificity, sensitivity, and efficiency [18]. Michaela Nesvadbova et al. [15] developed an integrated system of four multiplex qPCR assays that enabled the simultaneous quantitative detection of turkey, quail, duck, and goose-derived components.

Nevertheless, conventional multiplex TaqMan systems require target-specific primers and probes for each species, a requirement that increases design complexity and the risk of inter-primer competition, which may cause imbalanced amplification efficiencies. The use of universal primers can simplify the reaction system and fundamentally eliminate or reduce the possibility of potential primer-primer/primer-probe dimerization [19], which can effectively reduce the concentration of species-specific primers and create prerequisites for further improving the throughput of multiplex PCR assays [20]. In recent years, universal primer-based approaches combined with sequencing have been actively explored for species identification. Bertolini et al. [21] proposed a mitochondrial DNA (mtDNA)-based approach utilizing the Ion Torrent next-generation sequencing (NGS) platform, where universal primer pairs targeting conserved mtDNA regions were used to amplify and sequence mixed DNA from species such as chicken, duck, and goose. However, this approach struggled to meet the requirements for rapid and quantitative detection, as it still relied on time-consuming downstream sequencing steps. Consequently, there is an urgent need for novel methodologies that can achieve simultaneous, quantitative, and efficient detection of species-specific components in chicken, quail, duck, and goose products.

**Figure 1:**
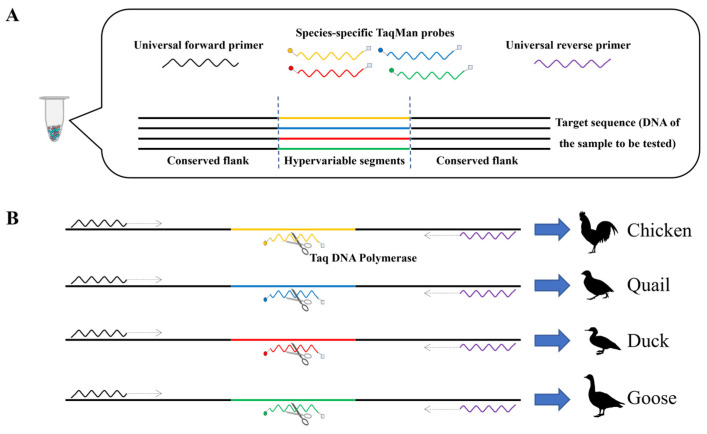
Schematic of the PUP-fPCR system. (**A**) PCR reaction system. (**B**) Components from different species emit different fluorescence.

This study aimed to identify a poultry-specific nuclear DNA (nDNA) sequence harboring conserved regions and interspecies polymorphic hypervariable segments. This sequence was then used to develop universal primers (targeting conserved flanks) and TaqMan probes (specific to hypervariable segments). Subsequently, a poultry universal primer-based fluorescent PCR (PUP-fPCR) method, by integrating universal primers with species-specific TaqMan probes, was established to accurately and efficiently quantify the content of chicken-, quail-, duck-, and goose-derived components in a single reaction (Figure 1). Notably, relative to conventional approaches, this method minimizes primer cross-reactivity, reduces probe design complexity, and markedly enhances assay throughput. Furthermore, by facilitating the streamlining of multi-species analysis, the technique has enriched the toolkit for animal species authentication and has provided robust technical support for regulatory agencies to strengthen market oversight and combat food adulteration.

## 2. Results and Discussion

### 2.1. Identification of nDNA Target Sequence, Poultry Universal Primers, and Species-Specific Probes for Chicken, Quail, Duck, and Goose DNA

**Figure 2:**
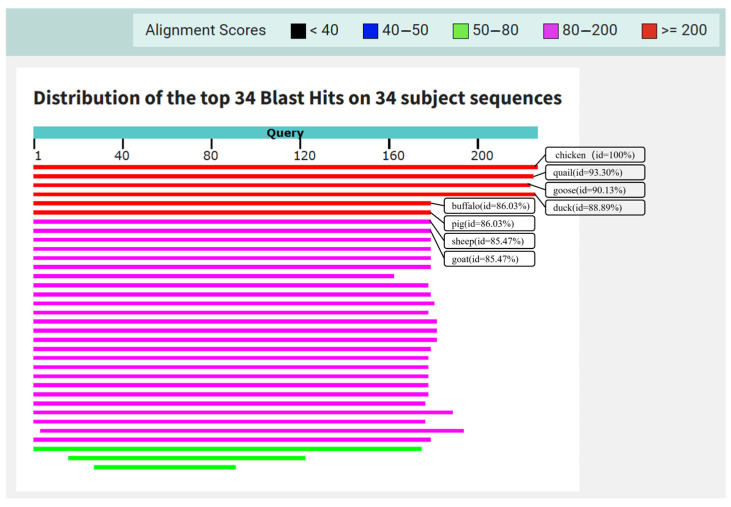
BLAST alignment results of the target sequence against the genomic sequences of 34 species.

**Figure 3:**
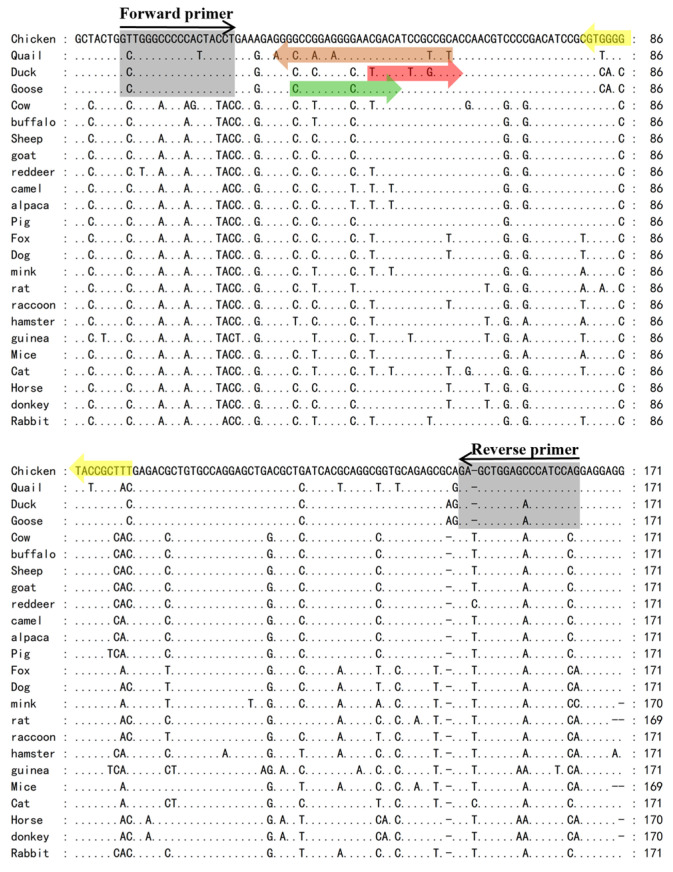
ClustalW multiple sequence alignment analysis (Multiple sequence alignment of nDNA sequences from 4 target species and 20 non-target species. Gray-shaded regions indicate universal primer-binding regions; regions shaded yellow, brown, red, and green correspond to the species-specific probe-binding regions for chicken, quail, duck, and goose, respectively. A dot “.” indicates a match with the reference sequence above, while a hyphen “-” indicates a base deletion.).

Through comparative screening of chicken nDNA sequences against whole genomes of other species, five exons were selected as candidate sequences (Table 1). After manual verification, exon 26 of the AMPD2 gene was identified as the target sequence for subsequent analysis. To assess its specificity, Nucleotide BLAST was performed against the NCBI reference genomes of four target poultry species (chicken, quail, duck, goose) and 20 non-target species. The results demonstrated that all target species met the predefined criteria for sequence alignment, including query length >200 bp, 85% ≤ identity ≤ 95%, and E-value < 10^−5^. In contrast, non-target species failed to meet these criteria, with query length < 200 bp, identity < 85%, or E-value ≥ 10^−5^. This comparison unequivocally confirmed the high species specificity of AMPD2 exon 26, validating its suitability for multi-species detection. High homology in the chicken (100%), quail (93.30%), goose (90.13%), and duck (88.89%) sequences ensured sufficient conserved regions for poultry universal primer design (Figure 2). To further evaluate primer/probe design feasibility, homologous sequences of the 20 non-target species were downloaded and aligned with the chicken AMPD2 exon 26 sequence. ClustalW multiple sequence alignment and Genedoc visualization were then applied to all 24 species (4 targets + 20 non-targets). This analysis yielded two critical findings supporting primer and probe design. First, conserved flanking regions existed exclusively across all four target poultry species but not in the other 20 non-target species, making them optimal for designing universal poultry-specific primers. Second, species-specific nucleotide variations in the internal region of the target sequence were unique to each of the four target poultry species, providing ideal targets for developing species-specific TaqMan probes (Figure 3). The selection of nDNA as the target molecule is predicated on its inherent stability and taxonomic specificity, which surpass those of mtDNA. On the one hand, although the multicopy nature of mtDNA facilitates amplification, its high mutation rate and inter-tissue variability in copy number may lead to false-negative results [22,23]. In contrast, nDNA within tissues and organs possesses a more stable structure [24]. Its conservation and fixed copy number ensure reliable quantitative analysis across diverse biological samples, making it particularly suitable for species identification applications [25]. On the other hand, mtDNA content varies across different tissue types; thus, mtDNA cannot be used to quantify species in unknown tissue samples unless the tissue type is confirmed in advance. These characteristics of nDNA form the methodological basis of this study, highlighting the rationale for prioritizing nDNA in the design of quantitative assays.

### 2.2. Evaluation of Specificity and Conservation of the Poultry Universal Primers and TaqMan Probes

**Figure 4:**
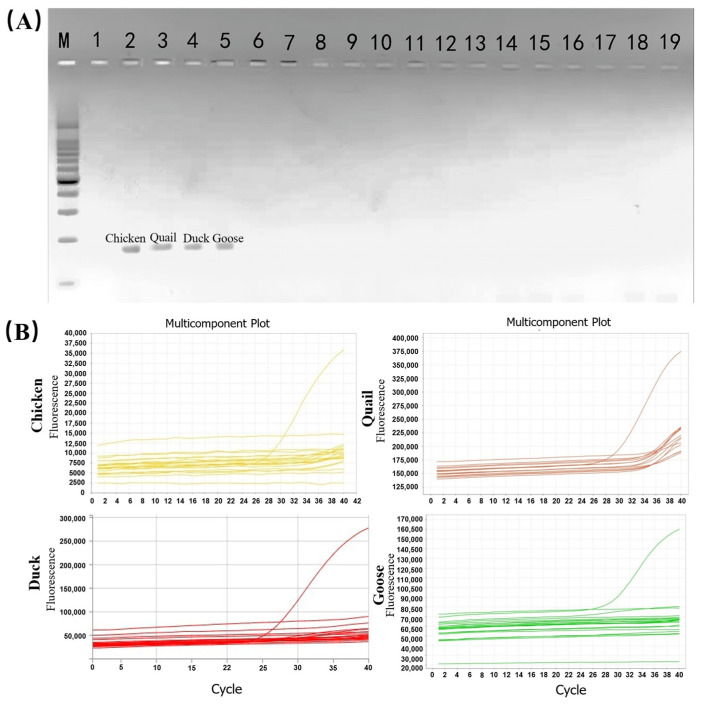
(**A**) Electrophoretogram of the poultry universal primer specificity assay. M: 50 bp DNA Ladder; lane 1: blank control; lanes 2–19: chicken, quail, duck, goose, rat, mouse, hamster, guinea pig, cow, sheep, pig, horse, rabbit, dog, fox, raccoon dog, cat, mink; (**B**) Specificity verification of TaqMan probes for chicken, quail, duck, and goose via singleplex real-time fluorescence PCR.

The specificity of primers and probes is a fundamental prerequisite for PCR-based species authentication assays [26]. In multiplex PCR, primer design is particularly challenging owing to the requirement for balanced specificity and conservation across all target amplicons [27]. In this study, to account for sequence variations among the four target species, degenerate bases were incorporated at two upstream and one downstream polymorphic sites within the poultry universal primers, thereby ensuring uniform amplification efficiency. Conventional PCR was performed to assess the quality of the universal primer pairs, using genomic DNA from the four target species (chicken, quail, duck, goose) and 14 non-target species commonly involved in meat adulteration. Agarose gel electrophoresis of the amplicons revealed specific bands of approximately equal intensity exclusively in the four target species, with no amplification observed in non-target templates or the blank control [Figure 4A]. These findings confirm the high specificity and conservation of the universal primers for poultry targets, with no cross-reactivity to other taxa. Subsequent validation of the species-specific TaqMan probes was conducted via singleplex real-time PCR across all target and non-target species. As shown in Figure 4B, each probe generated fluorescence signals solely in its corresponding target species, with no detectable amplification in non-target DNAs. This confirms the probes’ ability to discriminate between closely related poultry species with minimal cross-contamination. The species-specific TaqMan probes were designed to target hypervariable regions between conserved primer-binding sites, with each probe containing at least two species-specific nucleotide substitutions to enable unambiguous discrimination of target species via real-time fluorescence. This strategy leverages the 5′-nuclease activity of Taq polymerase, which cleaves the probes bound to complementary targets during amplification, releasing fluorophores from quencher proximity and generating species-specific signals [28]. Collectively, this validation not only ensured high reliability in detecting target poultry DNA but also effectively reduced the risk of false positives and non-target species interference, and verified the specificity of both poultry universal primers and species-specific TaqMan probes. This outcome laid a solid foundation for multiplexed quantitative analysis in complex sample matrices.

### 2.3. Establishment and Optimization of PUP-fPCR for the Detection of Chicken, Quail, Duck, and Goose

**Figure 5:**
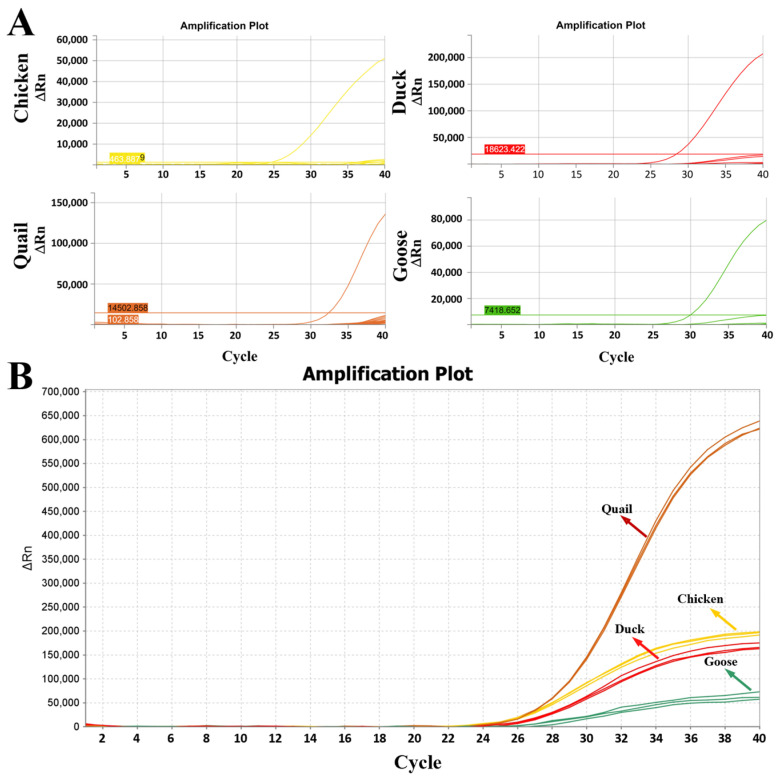
Establishment and optimization of the PUP-fPCR system. (**A**) Verification of the specificity of TaqMan probes for chicken, quail, duck, and goose via multiplex fluorescent PCR; (**B**) Simultaneous detection of chicken, quail, duck, and goose DNA in a single reaction using PUP-fPCR.

After profiling the fluorescence intensity of individual probes in singleplex assays and optimizing the multiplex PCR reaction system, optimal amplification was achieved with a probe ratio of 2:1:2:2 (chicken:quail:duck:goose). As shown in Figure 5A, specific fluorescence signals were detected exclusively in the presence of target species DNA, with no cross-reactivity observed for non-target animal components at Ct values <36. This confirms the high specificity of the multiplexed TaqMan system, where probes for the four target species exhibit no cross-amplification with non-target genomes. Additionally, the four distinct fluorescence amplification curves generated by the PUP-fPCR assay demonstrate that the method can simultaneously detect chicken, quail, duck, and goose in a single reaction (Figure 5B), while failing to detect the other 14 non-target species. Collectively, these results indicate that the PUP-fPCR system showed good specificity across 18 tested species.

Conventional multiplex PCR for multi-species identification requires designing species-specific primer pairs for each target, a cumbersome process that is prone to primer cross-reactivity and mismatches [19]. In contrast, real-time fluorescent qPCR offers high sensitivity, specificity, and throughput, making it an indispensable tool in food safety laboratories [29]. This study advances this technology by designing species-specific TaqMan probes targeting regions with species-specific internal nucleotide variations and combining them with poultry universal primers. The resulting PUP-fPCR method enables simultaneous identification and quantification of four poultry-derived components within a single reaction, while maintaining high specificity. In contrast to conventional multiplex PCR, this approach eliminates the need for post-amplification steps such as gel electrophoresis and Sanger sequencing, reducing turnaround time by approximately 50% while minimizing primer-primer competition. The optimized PUP-fPCR system demonstrates negligible cross-reactivity and enhanced detection efficiency, providing a robust technical solution for quality control of animal meat products.

### 2.4. Sensitivity of PUP-fPCR

**Figure 6:**
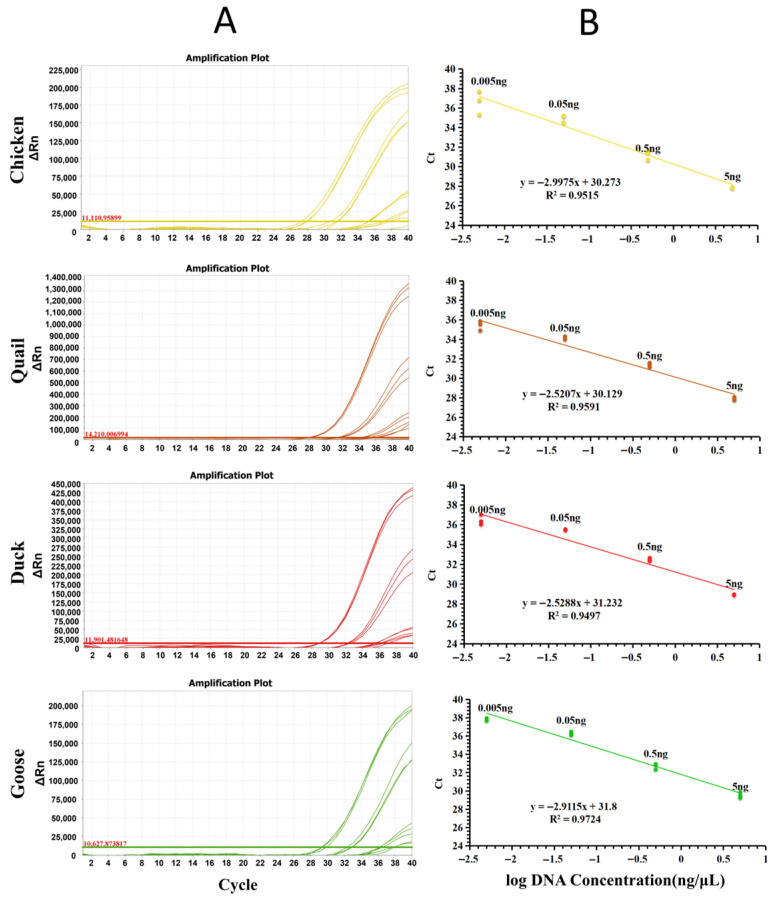
Amplification curves (**A**) and standard curves (**B**) of PUP-fPCR using serially 10-fold diluted DNA (5–0.005 ng/μL).

The limit of detection (LOD), defined as the lowest DNA concentration yielding a detectable fluorescent signal [18], was determined to evaluate the sensitivity of the PUP-fPCR assay. Genomic DNA extracted from chicken, quail, duck, and goose underwent 10-fold serial dilution (from 5 ng/μL to 0.005 ng/μL), with each dilution analyzed in triplicate. Sterile water served as the blank control in all experiments. As shown in Figure 6A, significant and stable fluorescence signals were detected at 0.005 ng/μL for all target species, thus establishing the LOD of the assay as 0.005 ng/μL. The sensitivity (LOD = 0.005 ng/μL) enables the detection of meat adulteration down to a level of 0.01% (weight/weight) [30]. The results proved that the method not only simplifies the reaction system but also maintains high sensitivity. Linear regression analysis was further performed to assess the assay’s quantitative performance. A robust negative correlation was observed between Ct values and log-transformed DNA concentrations across all four target species (R^2^ ≥ 0.94), with Ct values decreasing proportionally as the logarithmic DNA concentration increased (Figure 6B). This linear relationship indicates that the assay exhibits almost consistent amplification efficiency throughout its dynamic detection range. Previously, one study developed a multiplex PCR-based detection method capable of simultaneously detecting DNA from chicken, duck, and goose in commercial meat products, achieving a sensitivity of 0.05 ng [31]. Another study showed that qualitative real-time fluorescent PCR could detect chicken and duck components in meat, but it could not quantitatively determine the proportion of each species component in the meat [32]. In contrast, the PUP-fPCR method in this study elevated detection sensitivity to 0.005 ng, representing a tenfold enhancement over the universal primer-based quantitative method previously published by our team [19]. It not only enables simultaneous identification of four poultry species but also allows independent quantitative analysis of each component. This fully demonstrates its sufficient sensitivity and practical application value.

### 2.5. Quantification Analysis of DNA Mixtures Using PUP-fPCR

**Figure 7:**
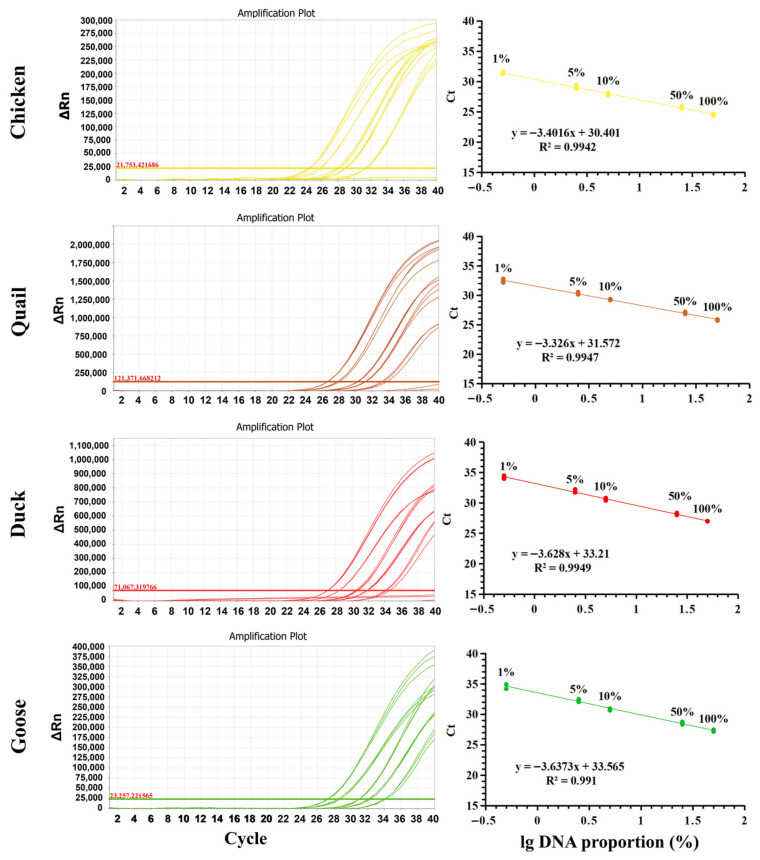
Standard curves for quantitative analysis of chicken, duck, goose, and quail DNA using PUP-fPCR assay (standard curves were generated using DNA samples with predefined proportions: 1%, 5%, 10%, and 100% of each target species DNA).

To evaluate the quantitative applicability of the PUP-fPCR assay, standard curves were constructed for chicken, quail, duck, and goose by plotting Ct values against log-transformed DNA concentrations. As shown in Figure 7, all standard curves exhibited strong linearity, with R^2^ of 0.9942, 0.9949, 0.9910, and 0.9947 for chicken, quail, duck, and goose, respectively. These R^2^ values exceed the acceptance criterion (R^2^ ≥ 0.98) established by the European Network of Genetic Laboratories (ENGL) for food authenticity testing [33], confirming the assay’s suitability for quantitative analysis. Accuracy and precision of the assay were further validated using DNA mixtures with predefined proportions of target species. Accuracy was assessed by calculating relative deviation (R.D.), while precision was determined via relative standard deviation (R.S.D.) [34]. Three composite DNA samples were prepared by spiking chicken, quail, duck, and goose DNA into pig DNA (a common non-target species in meat adulteration), and each sample was analyzed in triplicate. Since adulteration levels below 5% in meat products are considered economically unprofitable and thus are attributable to accidental contamination [35], the minimum DNA spiking level in the prepared composite samples was set at 5%. As summarized in Table 2, R.D. values for the composite samples ranged from 0.80% to 5.05%, and R.S.D. values ranged from 0.94% to 13.84%. Both metrics fall within the ENGL-recommended threshold (≤25%) [33], verifying the PUP-fPCR assay’s robust accuracy and precision for quantifying poultry-derived components in mixed matrices.

The development of the PUP-fPCR assay represents a notable methodological advancement for the simultaneous quantification of multiple poultry species in complex food matrices. Its performance, characterized by high linearity (R^2^ ≥ 0.9910) across all four target species, meets the stringent analytical standards set by ENGL for food authenticity testing [33]. This broad dynamic range ensures reliable quantification from trace-level contaminants (0.005 ng of DNA) to dominant components in mixed samples, addressing critical challenges in detecting both accidental cross-contamination and intentional adulteration. The low R.D. (0.80–5.05%) and acceptable R.S.D. (0.94–13.84%) observed in synthetic DNA mixtures align with international guidelines for quantitative PCR assays [33,34], validating the method’s utility in real-world scenarios where premium species like goose are often substituted with cheaper alternatives such as chicken.

Multiplex PCR is commonly used for multi-species identification in PCR-based methods. Traditional multiplex PCR allows simultaneous detection of multiple targets, but the coexistence of multiple primer pairs easily leads to competition and cross-reactions, and its reliance on gel electrophoresis limits it to qualitative analysis [5,9,16]. Although the combination of TaqMan probes and primers has enabled multiplex quantitative analysis [25,30], the use of multiple primer-probe sets inevitably leads to a more intricate and less stable reaction system. In recent years, researchers have adopted universal primer approaches to reduce system complexity, yet most current methods are still limited to qualitative analysis [36]. A key innovation of the PUP-fPCR lies in its integration of poultry universal primers and species-specific TaqMan probes, which overcomes the inherent limitations of traditional multiplex PCR, such as primer competition and cross-amplification risks [37]. We simultaneously achieved qualitative identification and quantitative analysis of four poultry species. By targeting conserved regions of nDNA for universal primer binding and hypervariable loci for species-specific probe hybridization, the assay achieves high-throughput detection (four species in a single reaction) and cost efficiency. It simplifies the assay components, thereby reducing the costs of primer and probe synthesis as well as the consumption of reaction reagents. The single-tube assay replaces multi-tube stepwise operations, reducing labor and time costs. The entire analysis can be completed within one and a half hours, improving the testing efficiency for batch samples. Notably, while the current study validated the PUP-fPCR assay using purified DNA templates, its performance in processed food matrices (e.g., cooked meats, canned poultry products) remains to be fully evaluated. Food processing steps (e.g., heat treatment, high-pressure processing) can introduce PCR inhibitors (e.g., humic acids, lipids) or induce DNA degradation, which may compromise amplification efficiency [38]. We used this method to perform quantitative analysis on samples of processed meat that had undergone high-temperature, high-pressure treatment. The results showed that neither the R.D. nor the R.S.D. fell within the thresholds recommended by ENGL (≤25%) [33]. Therefore, this method is suitable for the quantitative analysis of fresh samples. While it is not recommended for the quantitative analysis of highly processed samples, it can serve as an effective tool for their qualitative detection. Future research should focus on assessing the assay’s tolerance to these processing-related factors in complex matrices. Additionally, expanding the target species panel to include economically important poultry like turkey and pigeon would further enhance the assay’s utility for comprehensive food authenticity testing across global supply chains.

In conclusion, the PUP-fPCR assay developed in this study provides a reliable, efficient tool for the simultaneous quantification of multiple poultry species in meat products, addressing urgent needs in food safety regulation and consumer protection. Its innovative design—combining the conservation of universal primers with the spectral discrimination of TaqMan probes—establishes a benchmark for multiplex DNA-based analysis in food science, offering scalable solutions for quality control, traceability, and adulteration monitoring. Continued validation in real-world food matrices and expansion of the target species panel will solidify its role as a transformative technology for ensuring global food integrity.

## 3. Materials and Methods

### 3.1. Sample Preparation

Meat samples of chickens, quails, ducks, geese, cattle, sheep, horses, pigs, and rabbits were purchased from local markets. The tissues of mink, foxes, raccoon dogs, dogs, cats, rats, mice, hamsters, and guinea pigs were obtained from Huazhong Agricultural University.

### 3.2. DNA Extraction and Concentration Determination

Approximately 25 mg of each animal tissue sample (e.g., cattle, sheep, pigs, horses) was used for DNA extraction with the Universal Column Genome Extraction Kit (Cwbio, Beijing, China), following the manufacturer’s protocol. Extracted DNA was stored at −20 °C. DNA concentration and purity were assessed using a NanoDrop 2000 Spectrophotometer (Thermo Fisher Scientific, Wilmington, DE, USA). For each sample, 1 μL of DNA stock solution was loaded onto the sample pedestal to determine concentration and quality. Samples were considered valid if the DNA concentration exceeded 50 ng/μL, with A_260_/A_280_ ratios between 1.7 and 2.1 and A_260_/A_238_ ratios between 1.8 and 2.2. High-concentration samples were diluted to approximately 50 ng/μL with double-distilled water in new centrifuge tubes, and the dilution parameters were recorded. Stock DNA was stored at −20 °C for long-term preservation, and dilutions were kept at 4 °C for short-term use.

### 3.3. Screening of nDNA Target Sequences for Chicken, Quail, Duck, and Goose

Exon sequences derived from chicken nDNA were aligned against reference genome sequences of pig, cattle, sheep, horse, rabbit, mouse, guinea fowl, and turkey using the local BLAST+ tool (NCBI BLAST+ 2.12.0+). Sequences meeting the alignment criteria of query length > 200 bp, identity < 80%, and E-value <10^−5^ were initially selected. These sequences were subsequently aligned with genome sequences of quail, duck, and goose, with those satisfying the parameters of query length > 200 bp, 85% ≤ identity ≤ 95%, and E-value < 10^−5^ retained as candidate target sequences for species identification. These sequences were further analyzed via Nucleotide BLAST (https://blast.ncbi.nlm.nih.gov/Blast.cgi accessed on 8 February 2024) against NCBI reference genomes of 34 species, including 29 animals, 3 plants, and 2 microorganisms (chickens, quails, ducks, geese, cows, buffalo, sheep, goats, horses, donkeys, camels, red deer, alpacas, pigs, rabbits, mink, fox, raccoon dog, dogs, cats, rats, mice, hamsters, guinea pigs, frogs, hedgehog, fish, brown bear, snakes, wheat, rice, maize, Salmonella, and yeast). Subsequently, the top 24 species with high homology were selected and subjected to multiple sequence alignment using ClustalW (https://www.genome.jp/tools-bin/clustalw accessed on 15 February 2024). Visual analysis was then performed using Genedoc software (version 2.6.0.2). Sequences conserved in target species (chickens, quails, ducks, geese) but absent or with low homology in non-target species were selected. Manual curation identified sequences with conserved primer-binding sites at both ends and species-specific nucleotide variations in the internal region, serving as target sequences for the four poultry species.

### 3.4. Design of Poultry Universal Primers and Specific Probes

The poultry universal primers were designed using Primer-BLAST (https://www.ncbi.nlm.nih.gov/tools/primer-blast/, accessed on 9 March 2024) following standard primer-design guidelines. TaqMan probes for chickens, quails, ducks, and geese were developed by targeting mutational sites within the screened target sequences and between primer-binding regions. The details of the universal primers and specific probes are shown in Table 3.

### 3.5. Conventional PCR

Conventional PCR was performed using an Applied Biosystems™ Thermal Cycler (Thermo Fisher Scientific, USA) in 20 μL reactions containing: 1.6 μL dNTP Mixture, 2 μL 10× PCR Buffer (Mg^2^+ Plus), 50 ng DNA template, 0.4 μL of each primer (10 μM), 0.2 μL TaKaRa Taq polymerase (5 U/μL, TaKaRa, Dalian, China), and 15.4 μL sterile water. The cycling conditions were: 95 °C for 3 min (pre-denaturation); 36 cycles of 95 °C for 30 s (denaturation), 60 °C for 30 s (annealing), 72 °C for 15 s (extension); and a final extension step at 72 °C for 2 min. PCR products were cooled on ice and analyzed by agarose gel electrophoresis. A 4% agarose gel containing Lab Red DNA dye (Coolaber, Beijing, China) was prepared using 0.5× TBE buffer (Leagene, Beijing, China). Samples were loaded as follows: 4 μL of 50 bp DNA Ladder (Tiangen Biotech, Beijing, China) in the first well, and 5 μL of PCR product mixed with 1 μL loading buffer in subsequent wells. Electrophoresis was conducted at 150 V for 30 min using a Liuyi Biotechnology electrophoresis system (Beijing, China).

### 3.6. Real-Time PCR

Singleplex and multiplex fluorescent real-time PCR were performed on a QuantStudio™ 6 Flex System (Thermo Fisher Scientific, USA) and QuantStudio™ 6 Pro Flex System (Thermo Fisher Scientific, USA). For singleplex reactions, the 20-μL mixture included: 1 μL DNA template (50 ng/μL), 10 μL 2× GoldStar Probe Mixture (Low Rox) (CWBIO, Wuxi China), 0.4 μL of each primer, 3.5 μL probe (10 μM), and sterile water. Multiplex reactions contained: 1 μL DNA template, 10 μL 2× GoldStar Probe Mixture, 0.4 μL of each primer, and 3.5 μL of four probes (1 μL Chicken-P, 0.5 μL Quail-P, 1 μL Duck-P, 1 μL Goose-P), adjusted to a final volume of 20 μL with sterile water. Samples were protected from light during loading. The thermal profile was: 95 °C for 10 min (pre-denaturation); 40 cycles of 95 °C for 15 s (denaturation) and 61 °C for 20 s (annealing/extension). Each sample was run in triplicate, with non-template controls (NTCs) included to monitor contamination.

### 3.7. Standard Curves Construction

Two sets of standard curves were constructed for quantitative analysis. For the first set, genomic DNA of chickens, quails, ducks, and geese was serially diluted to 5 ng/μL, 0.5 ng/μL, 0.05 ng/μL, and 0.005 ng/μL (with sterile water as NTC) and used as templates to plot cycle threshold (Ct) values against the logarithm of DNA concentration. Trendline equations and coefficients of determination (R^2^) were calculated. For the second set, 50 ng/μL genomic DNA was diluted to 1%, 5%, 10%, 50%, and 100% (with 0% as NTC) with 50 ng/μL pig DNA to generate standard curves for quantifying DNA proportions in mixtures.

### 3.8. Quantitative Analysis of DNA Mixtures

Three DNA mixtures were used to validate the accuracy and precision of the PUP-fPCR. The percentage of each poultry species’ DNA in the mixtures was calculated using the standard curve equation y = ax + b, where y is the Ct value, and a and b are the slope and intercept, respectively. The formula for calculating DNA proportion (*C*) is:

*C* = 10^(y−b)/a^ × 100%

where *C* represents the percentage of chicken, quail, duck, or goose DNA in the mixture.

## 4. Conclusions

In this study, we developed a single-tube PUP-fPCR method for simultaneous identification and quantification of chicken, quail, duck, and goose in mixed meat samples, using one pair of poultry-specific nuclear DNA universal primers and four species-specific TaqMan probes. This method achieved a detection limit of 0.005 ng/μL for all four target species, with linear standard curves (R^2^ ≥ 0.99) across the measured concentration range. Validation using prepared DNA mixtures yielded relative deviations (R.D.) of 0.80–5.05% and relative standard deviations (R.S.D.) of 0.94–13.84%, both meeting ENGL-recommended standards (≤25%). The universal primer design largely avoids primer dimer formation, non-specific amplification, and cross-reactions inherent in conventional multiplex systems using multiple primer pairs. However, quantitative accuracy did not meet ENGL acceptance criteria when applied to mixed processed meat samples subjected to high-temperature and high-pressure treatment. Therefore, this method is recommended for quantitative analysis of fresh meat samples and qualitative screening of highly processed products. Future work will expand the target species range and improve quantitative precision and accuracy for processed meat samples. Overall, this study provides a practical tool for poultry meat identification in routine origin testing through its simplified single-tube design with universal primers.

## Figures and Tables

**Table 1:** Summary of candidate sequences suitable for developing the PUP-fPCR method.

Exon ID	Gene	Chr	Length (bp)	Gene Description	Sequence Identity to Other Target Species (%)
ENSGALE00010061152	*FZD8*	2	384	Frizzled class receptor 8	90.63~94.01
ENSGALE00010059626	*MOGS*	4	207	Mannosyl-oligosaccharide glucosidase	89.37~92.75
ENSGALE00010292250	*ZBTB7B*	25	349	Zinc finger and BTB domain containing 7B	92.84~93.12
ENSGALE00010292045	*AMPD2*	26	226	Adenosine Monophosphate Deaminase 2	88.89~93.30
ENSGALE00010285483	*JUND*	28	347	JunD proto-onco, AP-1 transcription factor subunit	87.33~89.94

**Table 2:** Quantification of chicken, quail, duck, and goose DNA in composite mixtures using PUP-fPCR assay.

DNA Mixtures	Species	Actual Proportion (%)	Detected Proportion (%)	Accuracy (R.D.) (%)	Precision (R.S.D.) (%)
I	Chicken	10	9.71	2.85	3.39
	Quail	10	9.53	4.74	4.79
	Duck	10	10.41	4.13	0.94
	Goose	10	9.76	2.39	7.85
	Pig	60	—	—	—
II	Chicken	5	5.25	5.05	3.74
	Quail	15	15.29	1.92	7.59
	Duck	25	24.64	1.45	1.01
	Goose	35	34.58	1.20	13.84
	Pig	20	—	—	—
III	Chicken	15	15.60	3.99	3.32
	Quail	15	14.88	0.80	3.83
	Duck	20	19.62	1.92	4.21
	Goose	20	19.52	2.41	0.96
	Pig	30	—	—	—

—: No components of this species were detected.

**Table 3:** Specifications of primers and probes.

Species	Designation	Oligo Sequences (5′ → 3′)
	Forward primer	GY * TGGGCCCCCAY * TACCT
	Reverse primer	CTGGATGGK * CTCCAGCTC
Chicken	Chicken-P	TAMRA-CGTGGGGTACCGCTTT-MGB
Quail	Quail-P	CY5-AGGCCCAGAAGGGAACGACATCCGTCGT-BHQ3
Duck	Duck-P	ROX-TGACATTCGGCGCAC-MGB
Goose	Goose-P	VIC-TGTCGTTGCCCTCCGGG-MGB

* Degenerate bases: Y = C/T, K = G/T.

## Data Availability

The original contributions presented in this study are included in the article. Further inquiries can be directed to the corresponding author.
